# Abrasive Wear Behavior of Batch Hot-Dip Galvanized Coatings

**DOI:** 10.3390/ma17071547

**Published:** 2024-03-28

**Authors:** Thomas Pinger, Marco Brand, Sonja Grothe, Gabriela Marginean

**Affiliations:** 1 ZINQ Technologie GmbH, An den Schleusen 6, 45881 Gelsenkirchen, Germany; thomas.pinger@zinq.com; 2 Institute of Mechanical Engineering, Westphalian University of Applied Sciences, Neidenburger Str. 43, 45897 Gelsenkirchen, Germany; sonja.grothe@w-hs.de (S.G.); gabriela.marginean@w-hs.de (G.M.)

**Keywords:** batch hot-dip galvanizing, wear behavior, abrasion, zinc, coating

## Abstract

In recent decades, batch hot-dip galvanized (HDG) steel has proven itself in practical applications due to the good corrosion resistance of its components. Despite the importance of the mechanical-load-bearing capacity of these coatings, the wear behavior has, so far, only been investigated very sporadically and not systematically, so a quantification of the wear behavior and statements on the mechanisms are vague. Therefore, two body wear tests with bonded abrasive grain were carried out. Varying the friction rolls, load, and total number of cycles, the wear behavior was investigated. The mass loss and the layer thickness reduction were measured at different intervals. After the test, the microstructure in the cross-section and the hardness according to Vickers (0.01 HV) were evaluated. The results showed that the wear behavior of HDG coatings against abrasive loads can be characterized with the selected test conditions. Initially, the applied load removed the soft η-phase. As the total number of cycles increases, the η- and ζ-phases deform plastically, resulting in a lower mass reduction compared to that expected from the measured layer thickness. The characteristic structure of a batch HDG coating with hard intermetallic Zn-Fe phases and an outer pure zinc phase has demonstrated effective resistance to abrasion.

## 1. Introduction

While the very first application of hot-dip galvanizing was in 1742, steel structures and components have been hot-dip galvanized (HDG) on an industrial scale for over 160 years and have efficiently protected against corrosion [1,2]. The coating is used in, e.g., building and road construction and the automotive and electrical industries [3]. On one hand, the corrosion resistance of zinc coatings is characterized by its passive protective properties, which lead to the formation of very stable, natural passive layers on the surface. On the other hand, zinc has a more negative electrochemical potential compared to steel, which protects the steel cathodically and, thus, actively protects it against corrosion [4,5].

**Figure 1:**
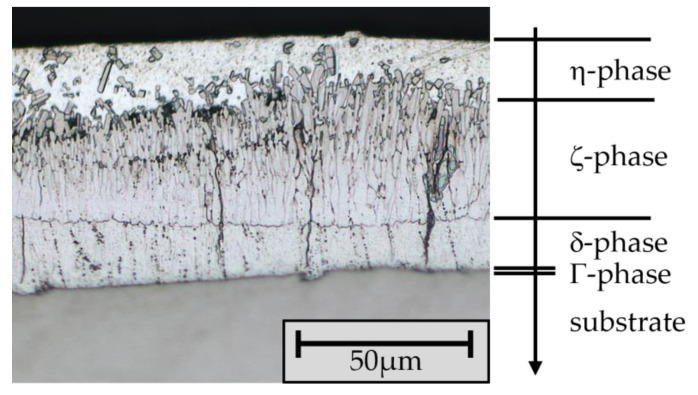
Typical structure of a component hot-dip galvanized in accordance with DIN EN ISO 1461 ([4], modified).

The zinc coating is applied after the appropriate wet chemical and/or mechanical cleaning of the steel component by immersing it in a zinc melt. According to DIN EN ISO 1461 [6], the melt has a zinc content of at least 98% and is operated at a process temperature of 445–455 °C. During the dwell time of the steel in the melt, usually 5–15 min, reciprocal diffusion processes take place between the metallic elements Fe and zinc, resulting in the formation of intermetallic Zn-Fe phases (Figure 1).

The Γ-phase directly adjacent to the substrate has a thickness of a few tenths of a micrometer and a relatively high Fe content (18–21%), which makes the phase very brittle. The subsequent δ-phase, with a hardness of up to 350 HV, is very compact, brittle, and uniform, with an Fe content of 7–11.5%. The ζ-phase (3.7 to 7.5% Fe) is characterized by distinct Zn-Fe dendrites, which are responsible for growth in the thickness of the coating. The phase is also brittle and has a hardness of up to 208 HV. The final η-phase is a pure zinc phase that is deposited on the Zn-Fe phases when the component is withdrawn from the zinc melt. Unlike the other phases, this phase contains little Fe (0.03%), which makes the phase very soft (50–70 HV) and ductile. In Table 1, an overview of the different phases and their properties is given. Depending on various factors, such as the reactivity of the steel, the dwell time in the zinc bath, or the zinc bath temperature, HDG coatings with a thickness of 50–250 µm are manufactured [7,8,9,10].

Regarding the behavior of HDG coatings when subjected to mechanical stress, their microstructure and adhesion are of particular importance. The metallurgical bond between the coating and the substrate is essential and ensures a very high load-bearing capacity of such galvanized steel components. Although this characteristic is widely known and appreciated in practice, suitable test methods have not been established or are not very specific, and studies to quantify the specific properties are rare. Accordingly, statements on the specific, scientifically substantiated properties of HDG coatings under various types of mechanical loads remain vague [6,9,10,11].

Regarding the adhesive strength of HDG coatings, DIN EN ISO 1461 states that this characteristic ‘does not normally need to be tested, as sufficient adhesive strength is characteristic of the galvanizing process, and the galvanized component should be able to withstand handling appropriate to the type and thickness of the zinc coating and the normal use of the component without peeling or flaking’ [6].

Systematic investigations by Katzung [11] using an adapted stamp pull-off test based on DIN EN ISO 4624 [12] show that the values achieved depend very strongly on the characteristics of the Zn-Fe phases. However, with values between ≈13 and ≈25 MPa for classic zinc melts, the results they achieved are considerably higher than those obtained with organic coatings or thermal-sprayed zinc coatings, for example. Such corrosion protection systems only bond to the substrate via adhesion forces. According to DIN EN ISO 12944-6 [13], such coatings have a minimum adhesive strength of 2.5 MPa; typical values are in the range of 3–10 MPa.

The high resistance of batch hot-dip galvanized coatings under mechanical load with values similar to those found by Katzung et al. [11] were also demonstrated by Abeln et al. [8] as part of investigations into the behavior of the adhesive joints of batch hot-dip galvanized components. Using thick shear tensile specimens and varying the zinc coating, the load-bearing capacity of the bonded joints under mechanical shear loading—and, thus, indirectly, also of the zinc coatings—was tested. According to DIN EN ISO 14713-2 [14], for steels of category A and B, i.e., those with a zinc coating characteristic as shown in Figure 1, an average shear strength of 14.5 MPa was demonstrated in the bonded joint. For category C and D steels, which have a much more distinctive ζ-phase, but no pure zinc (η) phase, failure occurred at 26.0 MPa on average. Across all tests, the failure of the cohesion of the zinc coatings was predominantly decisive for the load-bearing capacity of the bonded joints.

Corresponding test methods are known, considering the resilience of corrosion protection systems under a local impact load. DIN EN ISO 20567-1 [15] describes a stone impact test that was developed in the context of the automotive industry. As part of the test, the coated or galvanized test specimens are impacted with small, sharp-edged impact bodies at 45° and at a defined working pressure. The damage that occurs was assessed using reference samples. The characteristic value 1.5, which correlates with a damage of 2.5% of the surface [16], was demonstrated on thin-film galvanized components.

Wear describes the mechanical removal or reduction in material due to a wear body (e.g., sand), which can restrict or prevent the function of the component. In addition to material properties such as the microstructure [17], yield strength [17], or ductility [18], temperature [19], the phase transformation induced by the load [20], and the hardness ratio between the abrasive body and the surface to be tested [21] have an influence on the wear behavior. The behavior of zinc coatings under abrasive wear loads has been investigated in the past using various test devices.

Schröder [22] describes a method developed by the German Federal Waterways Engineering and Research Institute for testing corrosion protection coatings. In this method, coated test specimens are clamped in an octagonal testing machine, which then rotates when filled with various types of rock and, if necessary, water. The test specimens are, thus, tested for impact and abrasion stress.

In a study conducted by Szabadi et al. [23], the wear behavior of hot-dip galvanized steel sheets (S235JRG2) with loose abrasive particles (ballast stone 2–8 mm in diameter) was investigated. For this purpose, the samples were fixed to an ‘agitator’ (comparable to a conventional mixer), which was in a container with the abrasive bodies. Among other findings, it was found that the abrasion of the galvanized surface is not dependent on the rotational speed of the stirrer. As the test series was not based on a standardized procedure, the results are not reproducible.

Jędrzejczyk and Szatkowska [10] investigated the wear behavior of HDG coatings with and without heat treatment using the pin-on-disk test method. In this test method, a pin-shaped abrasive body (in this study, a steel pin) is contacted with the surface to be tested under a specific load. The sample then rotates at the defined number of cycles. The tested specimens were produced in accordance with PN EN ISO 10684 [24] and then post-treated at 200–530 °C in an oven. The results showed that suitable heat treatment can improve the wear behavior and increase the microhardness, whereas a heat treatment that is too high has a negative effect on the wear behavior. The heat-treated layer consists of only three Zn-Fe phases (Γ, δ, and ζ).

In another study, Jędrzejczyk and Skotnicki [9] compared the wear behavior of thermal diffusion zinc coatings according to PNEN ISO 17668 [25] with HDG zinc coatings (with and without heat treatment) using the pin-on-disc test method. As part of this investigation, it was found that the coefficient of friction and weight loss decreases with increasing hardness.

Against the background of a small number of studies to date on abrasion behavior and the limited depth of knowledge in this respect, the wear behavior of conventional HDG samples in accordance with DIN EN ISO 1461 was examined in several series of tests using a standardized test technique. For this purpose, a two-body (abrasive body and surface to be tested) wear test with bonded grain was used, and the wear behavior of the zinc coating and its microstructural changes under mechanical load were analyzed.

## 2. Materials and Methods

For the investigation, 10 metal sheets measuring 100 mm × 100 mm × 5 mm (structural steel) were hot-dip galvanized in accordance with the requirements of DIN EN ISO 1461 [6] and DASt guideline 022 [26]. The wear behavior of the specimens under an abrasive load was examined using the Taber Abraser 1700/1750 rotational abrasion tester (Taber Industries, North Tonawanda, NY, USA). This method simulates two-body wear with bonded grain and is standardized by different standards (DIN EN ISO 9352 [27], ASTM D 1044 [28], and DIN EN 438-6 [29]). As no specific requirements or specifications are given in the standards regarding the test conditions of galvanized samples, two different friction rolls were used to characterize the behavior of the samples. Two tools were used as abrasive bodies: elastically bonded rubber friction rolls with aluminum oxide (type CALIBRASE friction roll CS-10 from Taber Industries, North Tonawanda, NY, USA) and ceramic bonded friction rolls with silicon carbide particles (type CALIBRADE friction roll H-22 from Taber Industries, North Tonawanda, NY, USA). The latter has a higher hardness (the hardness of aluminum oxide is 1530–2120 HV, compared to the hardness of silicon carbide, which is 2510–2650 HV [30]), whereby a higher abrasion load is to be expected. The initial diameter of both friction roll types is 52 mm (minimum diameter 44 mm).

**Figure 2:**
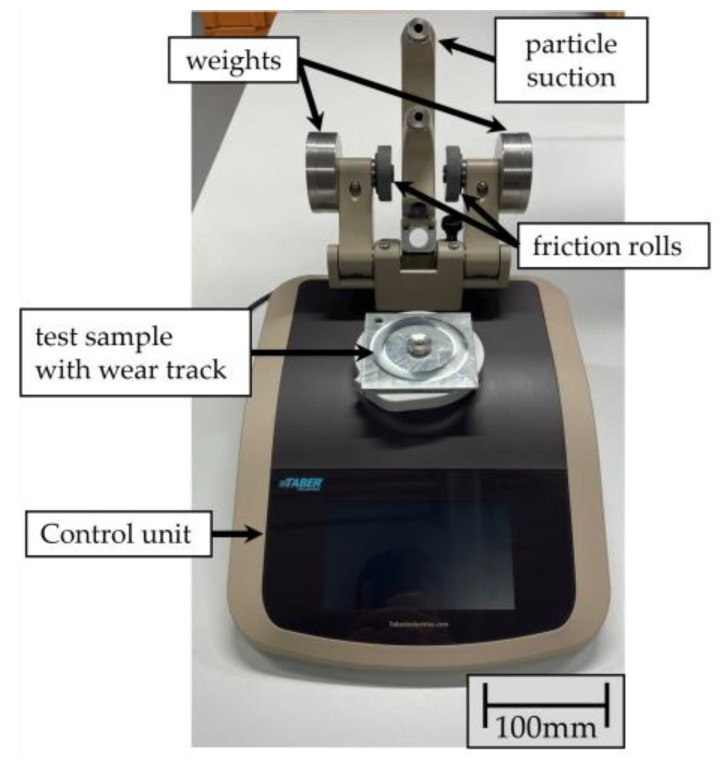
Test setup: Taber Abraser 1700/1750 rotational abrasion tester with samples to be tested.

For the test, the samples were first cleaned in an ultrasonic bath to remove transport-related impurities. The specimens to be tested were then clamped on the Taber, and the friction rolls were placed on the surface of the samples (see Figure 2). The rolls were loaded with additional weight to generate a defined contact pressure.

In the first series of tests with the CS-10 friction roller, a 500 g weight was selected. In the second series of tests, the weight was increased to 1000 g with the same friction rolls so that the influence of the weight could be observed. In both series, the specimens were tested for a total number of 1000 cycles. To simulate a particularly high mechanical load, in the third series of tests, the samples were loaded with a harder H-22 friction roll and a weight of 1000 g for a total number of 5500 cycles. In all tests, the rotation speed of the sample was set to 60 rpm. The friction rolls were pressed onto the specimen surface with the applied load. They can rotate in the setup, but are not driven. The wear particles produced during the test were continuously vacuumed. The test was stopped every 500 cycles to examine the condition of the specimens. The friction rolls were then dressed to ensure consistent quality of the friction rolls. The specimen was clamped back into the test device, and the test was continued.

**Figure 3:**
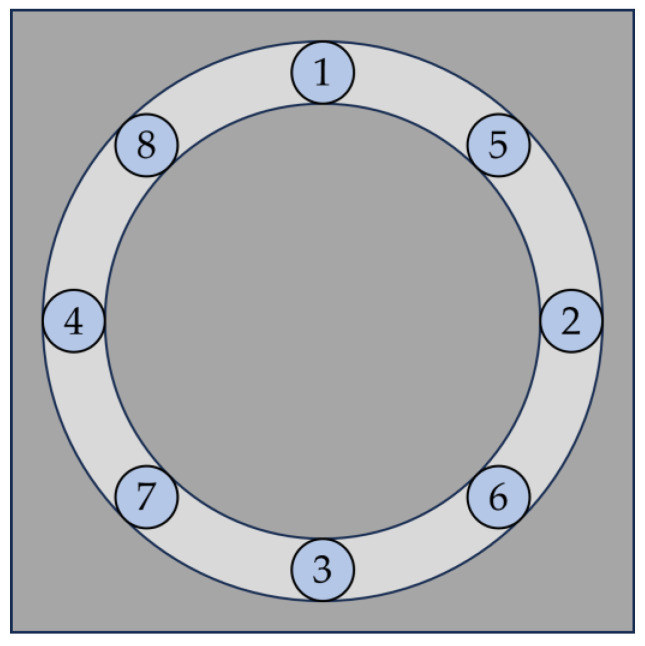
Measuring points to detect the remaining layer thickness in the wear track (1 at 0°, 2 at 90°, 3 at 180°, 4 at 270°, 5 at 45°, 6 at 135°, 7 at 225°, 8 at 315°).

The wear of the samples under abrasive load was investigated both gravimetrically and by measuring the coating thickness. The weight of each sample was measured in its initial state and after every 500 cycles using the Kern PNJ 600-3M precision balance (measurement uncertainty ±0.001 g; Kern & Sohn GmbH, Balingen-Frommern, Germany). The layer thickness was measured in parallel in accordance with DIN EN ISO 2178 [31] using the Erichsen LAYERCHECK 750 USB-FN universal probe (measuring uncertainty 2% + 2 µm; Erichsen GmbH & Co. KG, Hemer, Germany). This probe measures the layer thickness using the magnetic induction method in combination with the eddy current method. To consider process-related irregularities in the layer thickness, the respective layer thickness was determined as an average value at 8 measuring points with the aid of a template, and then the total average value was calculated from all measurements (see Figure 3). The measuring points are all located on the expected wear track.

In addition, cross-sections were made of individual samples to check the measured values and to characterize the coating in more detail. The cross-sections were prepared metallographically after cutting and then analyzed using a Hitachi Tabletop TM-3000 scanning electron microscope (Hitachi High-Technologies Corporation, Tokyo, Japan) and Zeiss Gemini Sigma 300 VP (Carl Zeiss AG, Oberkochen, Germany), respectively. To visualize the microstructure, the samples were etched in 0.5% HNO_3_.

The microhardness of the samples was measured perpendicular to the surface on the prepared cross-sections using the Zwick ZHVµ hardness testing machine (ZwickRoell GmbH & Co. KG, Ulm, Germany). The Vickers hardness was measured using a load level of 0.01 HV and a holding time of 10 s. To ensure that the individual measurements do not influence each other, a space of five times the indentation diagonal diameter was left between them. Determining the hardness of each individual phase, a total of 15 measurements were carried out on different specimens.

## 3. Results

**Figure 4:**
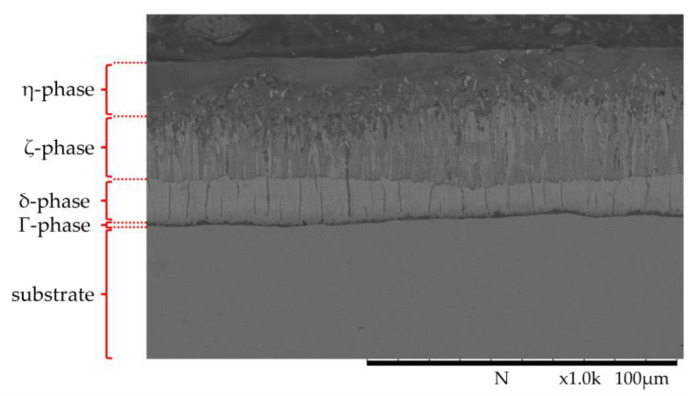
SEM image of HDG coating in mechanically unloaded condition.

The layer thickness of the zinc coatings of all samples was determined in the initial state using the magnetic inductive probe. The detected average layer thickness was 64.9 µm ± 8.49 µm. The characteristic structure with different phases is visible in the cross-section (Figure 4). The subdivision of the layer into the individual phases is clearly recognizable. The thin Γ-phase is directly adjacent to the substrate, followed by the δ- and ζ-phases and, finally, the η-phase.

Zinc coatings that are produced using the batch galvanizing process can exhibit fluctuations in coating thickness across the galvanized component, which are related to the process on one hand, and to the local scattering of the phase thickness on the other. During batch HDG, the immersion and extraction process inevitably results in a gradient of the dwell time and, thus, the coating formation time (first in, last out: the lower area of the steel component remains in the melt the longest, while the upper area remains in the melt the shortest). Furthermore, the flow behavior of the zinc over the component during extraction from the melt is not homogenous, which can affect the formation of the η-phase in particular. In addition, the formation of the Zn-Fe phases, especially the ζ-phase, is strongly dependent on the roughness and local reactivity of the steel, so the phase is not homogenously distributed, but it can vary in its specific thickness.

**Figure 5:**
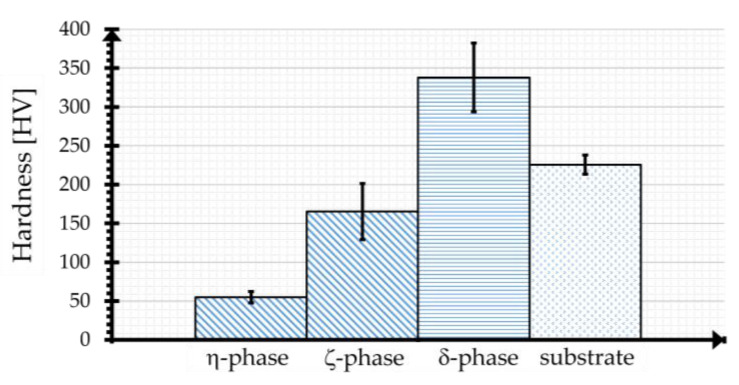
Mean hardness value (0.01 HV, 10 s) of unloaded zinc coating and substrate.

The different phases could be detected based on hardness measurements. As summarized in Figure 5, the η-phase has an average hardness of 55 HV ± 7.5 HV, whereas the δ- and ζ-phases are significantly harder at 165 HV ± 36.1 HV (ζ) and 338 HV ± 44.1 HV (δ), respectively, due to the higher Fe content of the respective phase. Due to the low thickness, it was not possible to measure the hardness of the Γ-phase with the testing technology used. As known from the literature, the hardnesses of the inter-metallic phases are higher than that of the substrate.

The abrasion test was then carried out according to the described measurement procedure, initially using the CALIBRASE CS-10 friction roll with a contact weight of 500 g or 1000 g for two steps with 500 cycles each. Table 2 shows the mean values of the mass losses resulting from five measurements and the reduction in the zinc layer thickness of the individual samples measured.

Both tests show a very uniform reduction in the coating mass over the two 500-cycle measurements. The measured layer thickness also decreases, whereby the values in the later cycles are each approx. 30% lower than those after the first 500 cycles.

**Figure 6:**
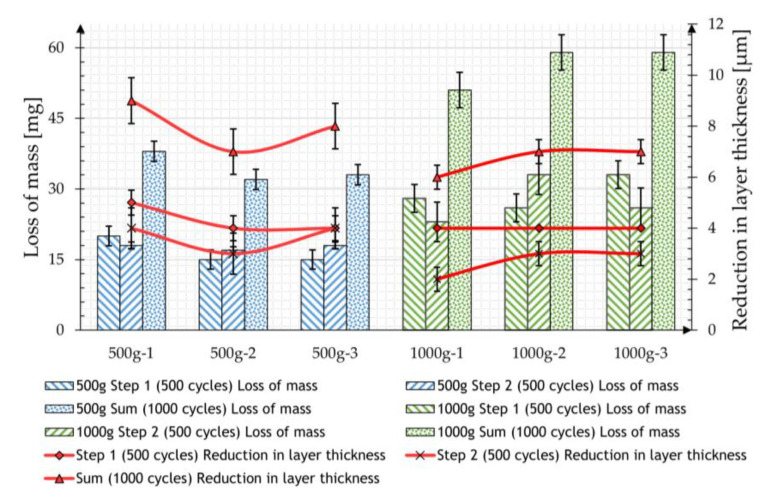
Mean loss of mass and mean reduction in layer thickness from specimens tested with CS-10 friction roll, 500 g load (blue), and 1000 g load (green).

On average, a greater loss of mass was observed in the second test due to the increased load. However, doubling the load only leads to a 60% increased material removal. The measured layer thicknesses in the case of the samples loaded with 1000 g show a smaller mass reduction than the samples loaded with 500 g, which is in contradiction to the recorded mass losses. It is noticeable that the removal detected in terms of mass is below the expected reduction in layer thickness of the samples. If a density of 7.2 g/cm^3^ [6] is assumed for the zinc coating, the measured removal should lead to a reduction of 3.6 µm in the case of the 500 g load and 5.8 µm in the case of the 1000 g load. The results of both tests are compared in Figure 6.

**Figure 7:**
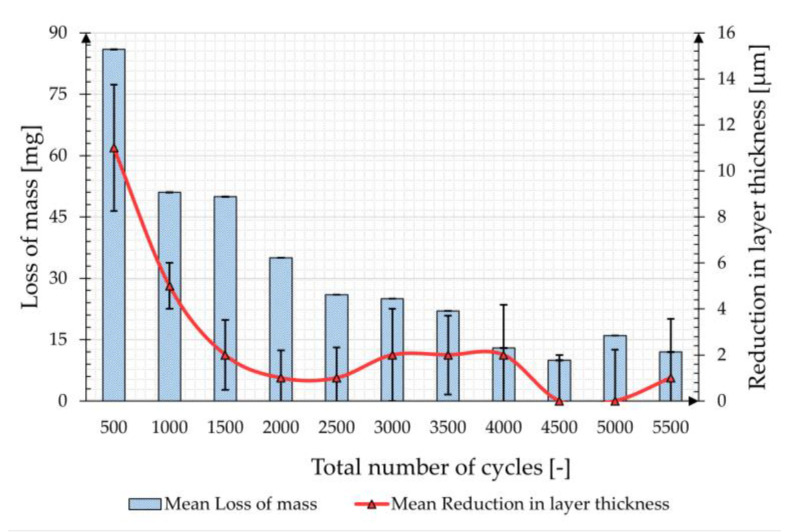
Mean loss of mass and mean reduction in layer thickness sample tested with H-22 friction roll and 1000 g load.

In the third series of tests, which was carried out with the ceramic-bonded CALIBRADE H-22 friction roll at a load of 1000 g, a duration with a total of 5500 cycles was applied to evaluate a particularly high load on the coating. Figure 7 shows the mean values of the mass losses and the reduction in layer thickness over the total number of cycles. The reduction in mass and layer thickness is higher within the first 500 cycles and decreases as the total number of cycles increases.

The evolution of the mass loss and layer thickness reduction match, although—as in the two previous test series—an almost continuous deviation between the two parameters can be observed. The total mass loss of the zinc coating after a total of 5500 cycles is 346 mg, which, assuming a density of 7.2 g/cm^3^ for the zinc coating, corresponds to a theoretical coating thickness of 36.01 µm. The cumulative coating thickness reduction, on the other hand, is 27 µm and is, therefore, approx. 25% lower than what would be expected from the weighing.

**Figure 8:**
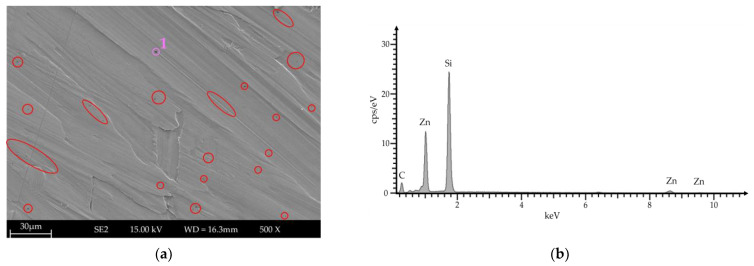
Top-view analysis of wear track. (**a**) SEM image with indented particles (highlighted in red) and (**b**) EDS analysis of indented particle at Position 1 in (**a**).

Top-view images were used to examine the wear track for indented particles. Existing indented particles were identified as silicon carbide using local energy dispersive X-ray spectroscopy (EDS; see Figure 8).

Based on the section shown, around 1.43% of the area examined is SiC particles. Assuming that this amount is constant over the entire wear path, the total weight of the particles was determined to be 0.462 mg based on the density of SiC (3.21 g/cm^3^). As the measurement uncertainty of the balance is 1 mg, the existing influence was classified as not relevant.

## 4. Discussion

Regarding the mechanical resistance of HDG coatings, their formation and characteristics are of essential importance. In this respect, the microscopic investigations were initially able to identify the characteristic development of the intermetallic zinc–iron phases with their representative hardness values, some of which are significantly higher than those of the steel substrate. The formation corresponds with the values known from the literature. In addition, the determined layer thickness varies widely depending on the measurement position. The upper areas of the sample tend to have thinner layer thicknesses than the lower areas. The difference is due to the dipping process and the associated pulling out of the samples, and is well-known in the literature and in practice.

The tests of simulated two-body wear with bonded grain on the galvanized specimens show the very high load-bearing capacity of this type of zinc coating. At the beginning of the tests, there is a constant mass reduction, which decreases as the total number of cycles increases. This behavior occurs both when using elastically bonded rubber friction rolls with aluminum oxide and when using ceramic bonded friction rolls with silicon carbide particles. Due to the large difference in hardness between the abrasive body and the hot-dip galvanized layer (top layer η-phase 55 HV), it can be assumed that the zinc layer is worn to a much greater extent, so a positive influence of the ductility of the η-phase on the wear behavior can be assumed. A comparable observation on the influence of ductility has already been observed by Kim and Kim on medium carbon steels [18]. They found that materials with similar hardness values exhibited different wear behavior; the higher the ductility, the better the wear performance.

The detected inconsistencies regarding the influence of contact pressure and the use of layer thickness as the test criteria compared to the removal of mass are striking. Contrary to expectations, doubling the load from 500 g to 1000 g does not lead to a linear increase in the removal of the zinc coating. Instead, there is only an increase of approx. 60%. Concerning the measured layer thicknesses, these deviate from the measured mass losses and the theoretical layer thickness reductions that can be derived from them. The order of magnitude is in the range of 20–30%. Even considering the general measurement inaccuracy of the applied method in the range of ±2% (see Section 2), as well as the roughness of the coating surface caused by the friction roller, the deviations can be described as significant and systematic.

**Figure 9:**
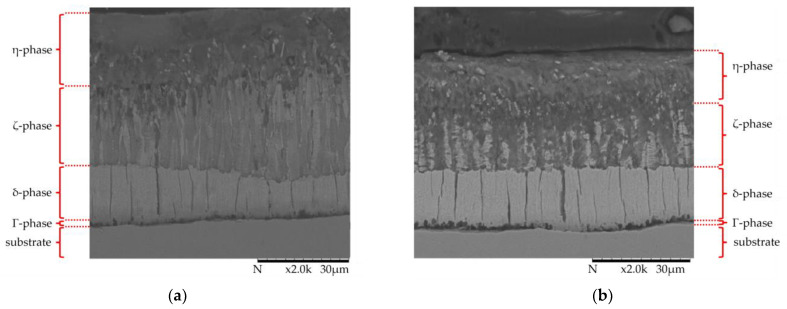
SEM cross-section of the etched specimen tested for a total of 5500 cycles (**a**) out of the track (unloaded area) and (**b**) in the track (loaded area).

To investigate this phenomenon, samples were analyzed in cross-sections following the series of measurements with the CALIBRADE H-22 friction roll. A comparison of the layer structure in the mechanically unloaded state next to the wear track and in the loaded test area after a total of 5500 cycles (in the wear track) is shown Figure 9.

The condition of the mechanically loaded zinc coating in the wear track differs significantly in the two upper phases (η- and ζ-phase). A comparison with the initial state shows that the thickness of the ductile soft η-phase, as well as the underlying ζ-phase, has been reduced. In addition to abrasive removal, the compaction of the two upper phases has apparently taken place. Both structures are plastically deformed and compacted; the transition area between the η- and ζ-phase is no longer clearly separated compared to the unloaded surface. The underlying δ-phase is unaffected compared to the original state, which can be explained by its higher hardness compared to that of the ζ-phase. This hard phase serves as a support structure for the upper softer phases, allowing them to press against each other and compress under a mechanical load.

**Figure 10:**
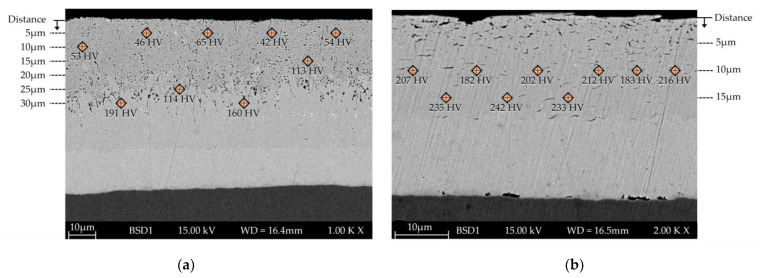
SEM cross-section of the specimen tested for a total of 5500 cycles with microhardness (0.01 HV, 10 s) values (**a**) out of the track (unloaded area) and (**b**) in the track (loaded area).

As compression causes an increase in hardness, the microhardness (0.01 HV, 10 s) was measured perpendicular to the surface to support the hypothesis. Figure 10 shows the unetched cross-section of a specimen in the wear track and out of the track in an unloaded area.

In the unloaded area, the different phases (η, ζ, and δ) are distinguishable from each other, with particulate phases in the uppermost layer. Depending on the distance from the surface, the hardness value corresponds to the characteristic values of an HDG coating. In contrast, as already mentioned, only two different areas can be differentiated within the loaded area. Resulting from the load, the η- and ζ-phase were supported on the δ-phase, which is why the phases are present in a vermicular structure. In addition, the hardness within this area is higher than the values detected for η and ζ outside the loaded area (inside the loaded area, hardness measurements closer than 10 µm to the surface did not result in valid values). The compression of the phases can be confirmed based on the changed phase distribution and the increased hardness. Such behavior has not been mentioned in the literature yet.

Regarding further systematic tests on the behavior of galvanized coatings under an abrasive load, the combination of a ceramic-bonded friction roll with silicon carbide particles with an additional 1000 g load and a total number of 2500 cycles is recommended. The softer friction rolls and/or a lower weight lead to significantly less material removal, which increases the testing effort without providing any additional knowledge. If possible, the gravimetric method should be preferred to the layer thickness measurement for evaluating the removal rate, as the latter can lead to distorted interpretations due to the compression effect of the upper phases.

The abrasive load removes parts of the zinc coating and plastically compresses the η- and ζ-phase. However, no damage or changes were detected in the remaining coating based on the tests we carried out. Based on this, no influence of the applied load on the properties of the HDG coating can be assumed. To examine this aspect in more detail, further investigations are planned regarding combined abrasion and corrosion stress in comparison to pure corrosion stress. The available knowledge about the specific behavior of HDG coatings and the existing interaction (e.g., regarding the steel-dependent formation of Zn-Fe phases, the combination of abrasion and corrosion stress, etc.) is not yet sufficient for the derivation of strategies to optimize wear resistance.

## 5. Conclusions

The high mechanical strength of hot-dip galvanized coatings is an essential property that ensures the durability of such galvanized products in a wide range of applications, some of which are very demanding. Although this characteristic is known and appreciated in practice, there are surprisingly few studies on abrasion-induced wear resistance that shed more light on this effect and quantify it. Accordingly, there is also a lack of concrete normative regulations. Therefore, several series of tests were carried out to investigate this fundamental property of HDG zinc coatings in more detail in accordance with DIN EN ISO 1461. For this purpose, the standard setup of the Taber test was used as a two-body wear test with bonded grain, and an adaptation to the specific behavior of zinc coatings was made by varying the test parameters (friction roller, load, and total number of cycles). To quantify the abrasion, the resulting mass loss and layer thickness reduction were recorded at continuous intervals and compared with the initial state. As a result, it was found that the selected test conditions can be used to characterize the wear of zinc coatings against abrasive loads. As the total number of cycles increases, the detected abrasion decreases, which can be attributed to the positive influence of the ductile η-phase in the normal loading case (500 g and 1000 g tested for a total of 1000 cycles). Under particularly high mechanical loads, a change in the coating structure was detected in the cross-section in terms of a plastic deformation and compaction of the η- and ζ-phase on the harder δ-phase.

A comparison of the selected evaluation methods shows deviations between the gravimetrically recorded mass losses and the magnetically inductively measured layer thickness reduction. For further systematic tests, the use of a ceramic-bonded friction roller with silicon carbide particles, an additional 1000 g load, a total number of 2500 cycles, and an evaluation of the output by recording the mass loss is recommended.

## Figures and Tables

**Table 1:** Phases of hot-dip galvanized coatings and their properties [8,9,10].

Phase	Thickness (µm)	Fe Content (Rest Zn) (%)	Hardness (HV)
Γ-phase	Few 0.1	18–21	Not determined
δ-phase	20–50	7–11.5	Up to 350
ζ-phase	Several 100	3.7–7.5	Up to 208
η-phase	Up to 100	0.03	50–70

**Table 2:** Mean loss of mass and mean reduction in layer thickness of measured specimens (CS-10 friction roll).

Load (g)	Total Number of Cycles (-)	Mean Loss of Mass per Step (mg)	Mean Reduction in Layer Thickness (µm)
500	500 (Step 1)	17.4 ± 2.06	4.6 ± 0.49
500	500 (Step 2)	17.2 ± 0.75	3.4 ± 0.8
500	1000 (Sum)	34.6 ± 2.15	8.0 ± 0.89
1000	500 (Step 1)	29.0 ± 2.94	4.0 ± 0.0
1000	500 (Step 2)	27.3 ± 4.19	2.7 ± 0.47
1000	1000 (Sum)	56.3 ± 3.77	6.7 ± 0.47

## Data Availability

Data are contained within the article.

## References

[1] Królikowska A., Komorowski L., Bonora P.L. (2020). Pitting Corrosion of Hot-Dip Galvanized Coatings. Materials.

[2] Kania H., Mendala J., Kozuba J., Saternus M. (2020). Development of Bath Chemical Composition for Batch Hot-Dip Galvanizing-A Review. Materials.

[3] Peng S., Xie S.-K., Xiao F., Lu J.-T. (2020). Corrosion behavior of spangle on a batch hot-dip galvanized Zn-0.05Al-0.2Sb coating in 3.5 wt.% NaCl solution. Corros. Sci..

[4] Pinger T., van den Bossche N. (2022). On the influence of zinc coating and outdoor exposure on the strength of adhesive, clinched, and hybrid joints of batch hot-dip galvanized steel. Int. J. Adv. Manuf. Technol..

[5] Pinger T. (2013). Stückverzinken–Ein Klassiker mit viel Potential. WOTech GbR.

[6] (2022). Hot Dip Galvanized Coatings on Fabricated Iron and Steel Articles—Specifications and Test Methods.

[7] Vourlias G., Pistofidis N., Stergioudis G., Tsipas D. (2004). The effect of alloying elements on the crystallization behaviour and on the properties of galvanized coatings. Cryst. Res. Technol..

[8] Abeln B., Pinger T., Richter C., Feldmann M. (2023). Adhesion of batch hot-dip galvanized components. Int. J. Adv. Manuf. Technol..

[9] Jędrzejczyk D., Skotnicki W. (2021). Comparison of the Tribological Properties of the Thermal Diffusion Zinc Coating to the Classic and Heat Treated Hot-Dip Zinc Coatings. Materials.

[10] Jędrzejczyk D., Szatkowska E. (2021). The Impact of Heat Treatment on the Behavior of a Hot-Dip Zinc Coating Applied to Steel During Dry Friction. Materials.

[11] Katzung W., Rittig R., Schubert P., Schulz W.-D. (1999). Haftfestigkeitsprüfung von Zinküberzügen mittels Abreißversuch in Anlehnung an DIN EN 24624. Metall.

[12] (2023). Paints and Varnishes—Pull-off Test for Adhesion.

[13] (2018). Paints and Varnishes—Corrosion Protection of Steel Structures by Protective Paint Systems—Part 6: Laboratory Performance Test Methods.

[14] (2020). Zinc Coatings—Guidelines and Recommendations for the Protection against Corrosion of Iron and Steel in Structures—Part 2: Hot Dip Galvanizing.

[15] (2017). Paints and Varnishes—Determination of Stone-Chip Resistance of Coatings—Part 1: Multi-Impact Testing.

[16] Pinger T. (2013). Dünnschicht-Stückverzinkungstechnologie als Ressourceneffizientes Korrosionsschutzsystem bei Verkehrsrückhaltesystemen. Straßenverkehrstechnik.

[17] Zambrano O.A., Gómez J.A., Coronado J.J., Rodríguez S.A. (2019). The sliding wear behaviour of steels with the same hardness. Wear.

[18] Kim S.-H., Kim Y.-S. (1999). Effect of ductility on dry sliding wear of medium carbon steel under low load conditions. Met. Mater..

[19] Okonkwo P.C., Kelly G., Rolfe B.F., Pereira M.P. (2012). The effect of temperature on sliding wear of steel-tool steel pairs. Wear.

[20] Nafar Dehsorkhi R., Sabooni S., Karimzadeh F., Rezaeian A., Enayati M.H. (2014). The effect of grain size and martensitic transformation on the wear behavior of AISI 304L stainless steel. Mater. Des..

[21] Viáfara C.C., Sinatora A. (2009). Influence of hardness of the harder body on wear regime transition in a sliding pair of steels. Wear.

[22] Schröder H.T. (1980). Abriebfestigkeiten der Korrosionsschutzbeschichtungen im Stahlwasserbau. Mater. Corros..

[23] Szabadi L., Kalácska G., Pék L., Pálinkás I. (2011). Abrasive wear of different hot-dip galvanized multilayers. Int. J. Sustain. Constr. Des..

[24] (2011). Standard—Fasteners, Zinc Coatings Applied by Hot-Dip Method.

[25] (2016). Zinc Diffusion Coatings on Ferrous Products—Sherardizing—Specification.

[26] Deutscher Ausschuss für Stahlbau (2016). DASt-Guideline 022. Guideline for Hot-Dip-Zinc-Coating of Load-Bearing Steel Components.

[27] (2018). Plastics—Determination of Resistance to Wear by Abrasive Wheels.

[28] (2019). Standard Test Method for Resistance of Transparent Plastics to Surface Abrasion by the Taber Abraser.

[29] (2016). High-Pressure Decorative Laminates (HPL)—Sheets Based on Thermosetting Resins (Usually Called Laminates)—Part 6: Classification and Specifications for Exterior-Grade Compact Laminates of Thickness 2 mm and Greater.

[30] Ullner C., Germak A., Le Doussal H., Morrell R., Reich T., Vandermeulen W. (2001). Hardness testing on advanced technical ceramics. J. Eur. Ceram. Soc..

[31] (2016). Non-Magnetic Coatings on Magnetic Substrates—Measurement of Coating Thickness—Magnetic Method.

